# Sustained therapeutic benefits by transient reduction of TDP-43 using ENA-modified antisense oligonucleotides in ALS/FTD mice

**DOI:** 10.1016/j.omtn.2023.01.006

**Published:** 2023-01-16

**Authors:** Toshihide Takeuchi, Kazuhiro Maeta, Xin Ding, Yukako Oe, Akiko Takeda, Mana Inoue, Seiichi Nagano, Tsuyoshi Fujihara, Seiji Matsuda, Shinsuke Ishigaki, Kentaro Sahashi, Eiko N. Minakawa, Hideki Mochizuki, Masahiro Neya, Gen Sobue, Yoshitaka Nagai

**Affiliations:** 1 Life Science Research Institute, Kindai University, 377-2 Ohnohigashi, Osaka-Sayama, Osaka 589-8511, Japan; 2 Department of Neurology, Kindai University Faculty of Medicine, 377-2 Ohnohigashi, Osaka-Sayama, Osaka 589-8511, Japan; 3 Department of Neurotherapeutics, Osaka University Graduate School of Medicine, Suita, Osaka 565-0871, Japan; 4 Department of Neurology, Osaka University Graduate School of Medicine, Suita, Osaka 565-0871, Japan; 5 KNC Laboratories Co., Ltd., Kobe, Hyogo 650-0047, Japan; 6 Department of Neurology, Nagoya University Graduate School of Medicine, Nagoya, Aichi 466-8550, Japan; 7 Department of Neurophysiology, National Institute of Neuroscience, National Center of Neurology and Psychiatry, Kodaira, Tokyo 187-8502, Japan

**Keywords:** MT: Oligonucleotides: Therapies and Applications, TDP-43, antisense oligonucleotides, amyotrophic lateral sclerosis, frontotemporal dementia, therapy, ENA, aggregation

## Abstract

The abnormal aggregation of TDP-43 into cytoplasmic inclusions in affected neurons is a pathological hallmark of amyotrophic lateral sclerosis (ALS) and frontotemporal dementia (FTD). Although how TDP-43 forms cytoplasmic aggregates and causes neurodegeneration in patients with ALS/FTD remains unclear, reducing cellular TDP-43 levels is likely to prevent aggregation and to rescue neurons from TDP-43 toxicity. To address this issue, here we developed gapmer-type antisense oligonucleotides (ASOs) against human TDP-43 using 2′-*O*,4′-*C*-ethylene nucleic acids (ENAs), which are modified nucleic acids with high stability, and tested the therapeutic potential of lowering TDP-43 levels using ENA-modified ASOs. We demonstrated that intracerebroventricular administration of ENA-modified ASOs into a mouse model of ALS/FTD expressing human TDP-43 results in the efficient reduction of TDP-43 levels in the brain and spinal cord. Surprisingly, a single injection of ENA-modified ASOs into TDP-43 mice led to long-lasting improvement of behavioral abnormalities and the suppression of cytoplasmic TDP-43 aggregation, even after TDP-43 levels had returned to the initial levels. Our results demonstrate that transient reduction of TDP-43 using ENA-modified ASOs leads to sustained therapeutic benefits *in vivo*, indicating the possibility of a disease-modifying therapy by lowering TDP-43 levels for the treatment of the TDP-43 proteinopathies, including ALS/FTD.

## Graphical abstract

**Figure:**
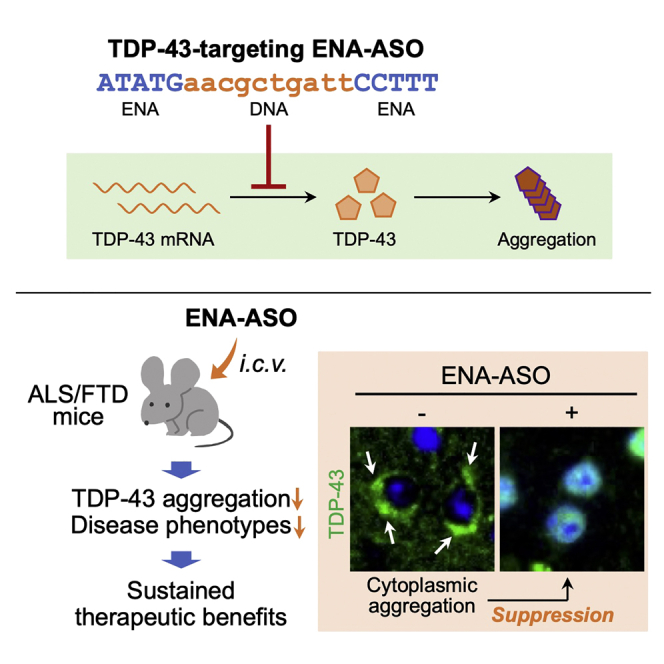

## Teaser

TDP-43 plays central roles in the pathogenesis of amyotrophic lateral sclerosis (ALS) and frontotemporal dementia (FTD). Takeuchi et al. demonstrate that the transient reduction of TDP-43 using gapmer-type antisense oligonucleotides suppresses cytoplasmic TDP-43 aggregation and disease phenotypes *in vivo*, indicating the therapeutic potential of the TDP-43-lowering approach for ALS/FTD treatment.

## Introduction

Amyotrophic lateral sclerosis (ALS) is a rapidly progressing neurodegenerative disease that is characterized by the selective loss of motor neurons in the brain and spinal cord, leading to paralysis and death within 5 years after clinical onset.^1^ A pathological hallmark of ALS is the abnormal aggregation into inclusions of TAR DNA-binding protein 43 (TDP-43), a DNA/RNA-binding protein localized primarily in the nucleus, accompanied by various post-translational modifications of TDP-43 such as ubiquitination, phosphorylation, and truncation in the cytoplasm of affected neurons. Almost all patients with ALS are thought to have TDP-43 pathology. This pathology is also observed in almost half of patients with frontotemporal dementia (FTD), which is currently recognized to be genetically, clinically, and pathologically in the same disease spectrum as ALS. Because TDP-43 plays essential roles in cellular activities, including nucleocytoplasmic transport, RNA processing, and stress granule metabolism, mislocalization and aggregation of TDP-43 are likely to cause abnormalities in various cellular functions, leading to detrimental consequences for cell survival.

Although how TDP-43 forms cytoplasmic aggregates and causes neurodegeneration in patients with ALS/FTD remains unclear, a reduction in cellular TDP-43 levels will likely prevent its aggregation and potentially rescue neurons from TDP-43 toxicity. Excess amounts of either wild-type (WT) or mutant TDP-43 was reported to cause neuronal toxicity and severe paralysis together with pathological TDP-43 aggregation in several lines of transgenic mice.^2^^,^^3^^,^^4^^,^^5^ Autophagy and the ubiquitin-proteasome system are important for TDP-43 clearance,^6^ and the induction of autophagy was shown to enhance TDP-43 degradation, leading to the suppression of TDP-43 aggregation and the alleviation of neurological disease phenotypes in a TDP-43 transgenic mouse,^7^ and in induced pluripotent stem cell-derived neurons with a pathogenic TDP-43 mutation.^8^ The targeted degradation of TDP-43 using single-chain antibodies was also shown to decrease TDP-43 levels and to effectively mitigate neuropathology in a mouse model of ALS/FTD expressing TDP-43.^9^ Importantly, using inducible TDP-43 transgenic mouse models, shutdown of TDP-43 expression after disease onset was shown to result in a robust reduction in TDP-43 pathology as well as in the functional recovery of disease phenotypes, including motor deficits and shortened life span.^10^^,^^11^ These results strongly indicate that the neuropathology and disease phenotypes of ALS/FTD can be reversed by lowering TDP-43 levels, at least at an early stage of disease progression. Although the complete elimination of TDP-43 is probably not appropriate because of its importance in cellular activities,^12^^,^^13^^,^^14^ lowering the level of TDP-43 is expected to be a potential disease-modifying therapeutic strategy to prevent the onset of or to delay the progression of ALS/FTD.^15^

Antisense oligonucleotides (ASOs) are emerging as an attractive therapeutic strategy for neurological diseases. ASOs are designed to bind disease-associated target genes or their transcripts, leading to modulation of the expression of target genes. After the first successful approval of the molecularly targeted ASO therapy (nusinersen) for spinal muscular atrophy,^16^ ASO-based therapies targeting disease-associated genes have been extensively investigated for the treatment of various neurological diseases, such as Huntington’s disease (HD), spinocerebellar ataxia type 3 (SCA3), Parkinson’s disease, and Alzheimer’s disease. In ALS/FTD, ASOs targeting disease-associated genes such as *SOD1*,^17^ *ataxin-2*,^18^ *FUS*,^19^ and *C9orf72*-associated repeat^20^^,^^21^^,^^22^ have been investigated and are now being clinically tested for their efficacy and safety in patients. However, ASOs that target TDP-43 have not yet been investigated for their possible therapeutic use^23^ and, therefore, the therapeutic potential of the TDP-43-lowering approach using ASOs remains unknown despite the central roles of TDP-43 in the pathogenesis of ALS/FTD.^24^

To address this issue, in this study we developed gapmer-type ASOs against human TDP-43 using 2′-*O*,4′-*C*-ethylene nucleic acids (ENAs), which are modified nucleic acids with high stability, and tested the therapeutic potential of the TDP-43-targeting approach using ENA-modified ASOs. We demonstrated that intracerebroventricular administration of ENA-modified ASOs into a mouse model of ALS/FTD expressing human TDP-43 results in the efficient reduction of TDP-43 levels in the brain and spinal cord. Surprisingly, a single injection of the ENA-modified ASO into TDP-43 mice led to long-lasting improvement of behavioral abnormalities and suppression of the mislocalization/cytoplasmic aggregation of TDP-43 even after the levels of TDP-43 had returned to the initial levels. Our results demonstrate that lowering TDP-43 levels using ENA-modified gapmer ASOs leads to the sustained improvement of disease phenotypes and TDP-43 pathology *in vivo*, indicating the therapeutic potential of the TDP-43-lowering approach for treatment of the TDP-43-associated diseases (TDP-43 proteinopathies), including ALS/FTD.

## Results

### Design and selection of ENA-modified gapmer ASOs that target endogenous human TDP-43

**Figure 1:**
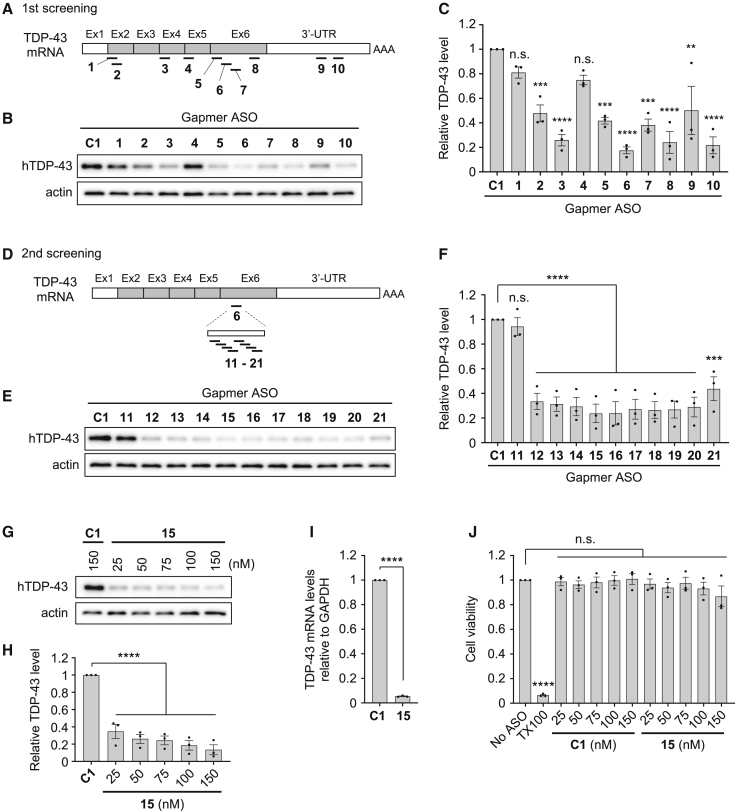
Screening and validation of gapmer ASOs that target human TDP-43 using HEK293 cells (A) Schematic representation of the target sites of gapmer ASOs **1** to **10** in TDP-43 mRNA. Ex, exon; UTR, untranslated region; AAA, poly(A) tail. (B and C) Western blot image (B) and bar graph (C) showing relative hTDP-43 levels in HEK293 cells transfected with control ASO **C1** or ASOs **1** to **10** (concentration, 75 nM; incubation, 48 h). Actin was used as a loading control in (B). (D) Schematic representation of target sites of gapmer ASOs **11** to **21**, which were designed based on ASO **6** in exon 6 of TDP-43 mRNA. (E and F) Western blot image (E) and bar graph (F) showing relative hTDP-43 levels in HEK293 cells transfected with ASO **C1** or ASOs **11** to **21** (concentration, 75 nM; incubation, 48 h). (G and H) Western blot image (G) and bar graph (H) showing relative hTDP-43 levels in HEK293 cells that were transfected with ASO **C1** (150 nM) or various concentrations of ASO **15** (25–150 nM), and incubated for 48 h. (I) Bar graph showing mRNA levels of TDP-43 in HEK293 cells transfected with either ASO **C1** or ASO **15** (concentration, 75 nM; incubation, 48 h), measured by qRT-PCR analysis. The mRNA levels of GAPDH were also measured and used for normalization. (J) Bar graph showing the viability of HEK293 cells transfected with ASO **C1** or ASO **15** (concentration, 25–150 nM; incubation, 48 h). Cells treated with 1% Triton X-100 were used as positive control. Data in (C), (F), (H), (I), and (J) are presented as the mean ± SEM of three independent experiments. Statistical analyses were performed to assess differences from the control group by one-way ANOVA followed by the Dunnett multiple comparisons test in (C), (F), (H), and (J), and Student’s t test in (I). ∗∗p < 0.01, ∗∗∗p < 0.001, ∗∗∗∗p < 0.0001; n.s., not significant.

To obtain proof of concept of the potential of the TDP-43-lowering approach for ALS/FTD treatment, we utilized gapmer-type ASOs with modified nucleic acids. Gapmer ASOs are short ASOs in which a DNA segment is placed at the center and flanked on both sides by RNA segments. Gapmer ASOs bind to their target RNAs and form a DNA:RNA hybrid, which can be recognized and cleaved by ribonuclease H, leading to silencing of the target gene. For the RNA segments in gapmer ASOs we utilized ENAs, which are modified nucleic acids with an ethylene bridge between the 2′-*O* and 4′-*C* positions of furanose.^25^ This ethylene linkage, which makes the furanose ring fixed in the *N*-type conformation, enables ENAs to interact with their complementary RNAs with comparably high affinity as other bridged nucleic acids (BNAs), such as 2′-*O*,4′-*C*-methylene-bridged nucleic acid/locked nucleic acid (2′,4′-BNA/LNA), but provides substantially higher resistance against nuclease degradation than 2′,4′-BNA/LNA.^26^ We used gapmer ASOs of 20 nt in length, in which ten nucleotides of DNAs are flanked by five nucleotides of ENAs on both sides, and a phosphorothioate linkage was included in the backbone for additional stability. The modulation of cellular TDP-43 levels is considered to be difficult owing to an autoregulatory feedback loop that maintains TDP-43 expression at a constant level.^27^^,^^28^ Therefore, we first designed ENA-modified ASOs that specifically target human TDP-43, based on previous reports in the literature of the successful reduction of TDP-43 levels using small interfering RNAs (siRNAs) in cultured cells or in mice, and performed cell-based screening of these ASOs to validate their knockdown efficiency. Table 1 shows the sequences designed in the first screening, which together cover almost the entire TDP-43 mRNA sequence, including the exons and 3′ UTR (Figure 1A). All gapmer ASOs were synthesized by solid-phase oligonucleotide synthesis based on phosphoramidite chemistry and were used at a purity of higher than 95%.

**Table 1:** Gapmer ASOs used in the first screening

ASO ID	Sequence^a^	Target	References
**C1**	CCTATaggactatccAGGAA	–	Jiang et al.^21^
**1**	GGGCTcatcgttctcATCTT	exon 2	Ayala et al.^12^, Iguchi et al.^13^, Aulas et al.^29^
**2**	TTCAAtgggctcatcGTTCT	exon 2	Ayala et al.^12^, Iguchi et al.^13^, Aulas et al.^29^
**3**	CAGTCttaagatcttTCTTG	exon 4	Koyama et al.^30^
**4**	CAAAGgctcatcttgGCTTT	exon 5	Aulas et al.^29^, McDonald et al.^31^, De Conti et al.^32^
**5**	TTAATgatcaagtccTCTCC	exon 6	Stoica et al.^33^
**6**	ATATAtgaacgctgaTTCCT	exon 6	Swarup et al.^34^
**7**	GTGCTtaggttaggcATTGG	exon 6	Iguchi et al.^13^
**8**	ATCCAtgcttgagccAAAGC	exon 6	Yu et al.^35^
**9**	AAGGCttcatattgtACTTT	3′ UTR	Lagier-Tourenne et al.^36^
**10**	AATATccattatgcaCCACC	3′ UTR	Yu et al.^35^

a Capital letter, ENA; small letter, DNA.

For evaluation of the knockdown efficiency of gapmer ASOs against TDP-43, the ASOs were transfected into human embryonic kidney (HEK) 293 cells, and 48 h post transfection the cellular levels of endogenous TDP-43 were analyzed by western blotting. Densitometric analysis demonstrated that most of the gapmer ASOs, except for ASOs **1** and **4**, decreased TDP-43 levels compared with the nontargeting control ASO **C1** (Figures 1B and 1C). This result demonstrates that most of the target sequences used for siRNA-mediated knockdown can also be used for gapmer ASO-mediated knockdown, despite the different knockdown mechanisms used by siRNAs and gapmer ASOs. Among the tested gapmer ASOs, ASO **6** showed the highest efficiency of TDP-43 knockdown, and ASOs **3**, **8**, and **10** modestly reduced TDP-43 levels.

We then focused on ASO **6** to optimize the target sequence of the gapmer ASOs. For this purpose, we further synthesized the gapmer ASOs **11–21**, in which the sequences were designed to be shifted by two bases each from the target sequence of ASO **6** (identical to ASO **16**), and the evaluation of TDP-43 knockdown was repeated using HEK293 cells (Figure 1D and Table 2). Among ASOs **11–21**, ASOs **12–20** showed knockdown efficiency comparable with that of the original ASO **6** (ASO **16**) (Figures 1E and 1F). As ASO **15** appeared to have the highest efficiency, we decided to use gapmer ASO **15** in the subsequent experiments.

**Table 2:** Gapmer ASOs used in the second screening and in the *in vivo* experiments

ASO ID	Sequence^a^
**C1**	CCTATaggactatccAGGAA
**11**	GCTGAttcctttaatGATCA
**12**	ACGCTgattcctttaATGAT
**13**	GAACGctgattccttTAATG
**14**	ATGAAcgctgattccTTTAA
**15**	ATATGaacgctgattCCTTT
**16**^b^	ATATAtgaacgctgaTTCCT
**17**	GGATAtatgaacgctGATTC
**18**	TTGGAtatatgaacgCTGAT
**19**	CATTGgatatatgaaCGCTG
**20**	GGCATtggatatatgAACGC
**21**	TCGGCattggatataTGAAC
**C2**^c^	CTTCTatcgtcgaatTTAGA

a Capital letter, ENA; small letter, DNA. The sequences identical to the DNA segment of ASO **6** (**16**) are underlined.

b The sequence of ASO **16** is identical to ASO **6**.

c Control gapmer ASO for the *in vivo* experiments designed based on the sequence of **15**, in which the nucleic acid composition of DNA and ENA is identical to **15** but the sequence is scrambled.

### Gapmer ASO **15** decreases TDP-43 expression without any toxicity

We evaluated the knockdown efficiency of ASO **15** against human TDP-43, as well as its potential cytotoxicity. HEK293 cells were transfected with various concentrations of ASO **15** and incubated for 48 h. Western blotting analysis after a 48-h incubation demonstrated that the protein level of endogenous TDP-43 was markedly reduced in the tested concentration range of ASO **15** of 25–150 nM (Figures 1G and 1H). qRT-PCR analysis showed almost complete knockdown of TDP-43 at the mRNA level, when cells were incubated for 48 h after transfection with 75 nM ASO **15** (Figure 1I). Treatment of cells with ASO **15** had no detrimental effects on their viability in the concentration range that we used for the reduction of TDP-43 expression, i.e., 25–150 nM (Figure 1J). These results collectively indicate that gapmer ASO **15** strongly suppresses TDP-43 expression at both the protein and mRNA levels without any apparent acute cytotoxicity.

### Gapmer ASO **15** reduces human TDP-43 levels in hTDP-43 mice

**Figure 2:**
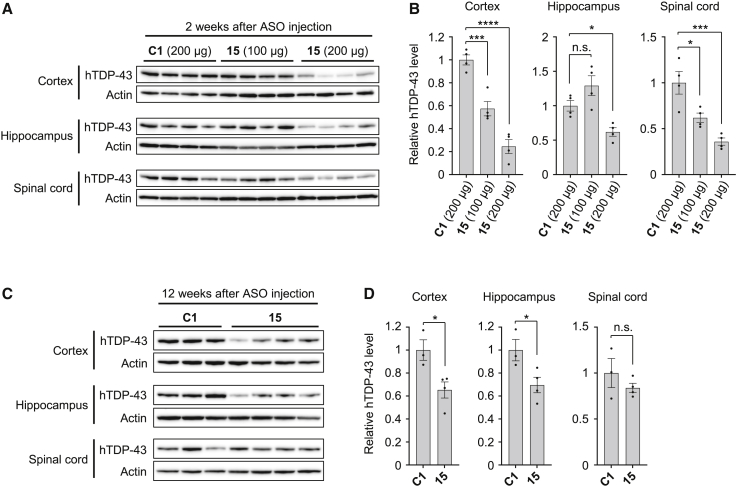
Intracerebroventricular administration of gapmer ASO **15** decreases TDP43 protein levels in the brain and spinal cord of hTDP-43 mice Western blot images (A and C) and bar graphs (B and D) showing relative hTDP-43 levels in hTDP-43 mice that were injected with either control ASO **C1** (200 μg) or ASO **15** (100 or 200 μg in A and B; 200 μg in C and D) into the cerebral ventricles at 6 weeks of age. Protein levels of hTDP-43 in the cortex, hippocampus, and spinal cord were analyzed at 2 weeks (A and B) and 12 weeks (C and D) after ASO injection. Actin was used as a loading control. Data in (B) and (D) are presented as the mean ± SEM of 3–4 mice. Statistical analyses were performed to assess differences from the control group by one-way ANOVA followed by the Dunnett multiple comparisons test in (B) and Student’s t test in (D). ∗p < 0.05, ∗∗∗p < 0.001, ∗∗∗∗p < 0.0001; n.s., not significant.

We next analyzed whether ASO **15** decreases the expression level of human TDP-43 *in vivo*. For this purpose, a mouse model of ALS/FTD expressing human TDP-43 with the familial mutation A315T (hTDP-43 mice)^37^ was used. hTDP-43 mice were generated by introducing a human TDP-43(A315T)-encoding gene fragment using a bacterial artificial chromosome. The resulting mice demonstrated not only cytosolic mislocalization and aggregation of TDP-43 in their neurons but also behavioral phenotypes associated with ALS/FTD.^37^ We injected either gapmer ASO **15**, or control ASO **C1** into the cerebral ventricles of hTDP-43 mice at 6 weeks of age and analyzed the expression levels of hTDP-43 using a human TDP-43-specific antibody (clone 2E2-D3) at 2 weeks, 12 weeks, and 24 weeks after injection. We confirmed that the administration of both gapmer ASO **15** and control ASO **C1** by intracerebroventricular (i.c.v.) injection was well tolerated and demonstrated no acute toxicity up to a dose of 200 μg of ASOs per mouse. Injection of ASO **15** at a dose of 200 μg resulted in a marked reduction in hTDP-43 levels, not only in the brain including the cortex and hippocampus, but also in the spinal cord 2 weeks after injection, whereas injection of ASO **15** at a dose of 100 μg modestly decreased hTDP-43 levels in the cortex and spinal cord but not in the hippocampus (Figures 2A and 2B). These results indicate that ASO **15** can reduce hTDP-43 expression *in vivo* and that a dose of 200 μg is sufficient to cause a marked reduction in hTDP-43 expression throughout the brain and spinal cord. Even at 12 weeks after injection, administration of ASO **15** at a dose of 200 μg still showed a modest reduction in hTDP-43 expression in the cortex and hippocampus, but not in the spinal cord (Figures 2C and 2D). However, a reduction in hTDP-43 levels was no longer detectable 24 weeks after injection (Figure S1). Collectively, significant reduction of hTDP-43 expression can be achieved by a single i.c.v. injection of ASO **15** at a dose of 200 μg and is sustained in the brain for at least 12 weeks after the injection.

In contrast, the administration of gapmer ASO **15** did not substantially affect the level of endogenous mouse TDP-43 mRNA in the cortex, hippocampus, and spinal cord of hTDP-43 mice at 2 weeks and 12 weeks after injection (Figure S2), indicating that gapmer ASO **15** targets human TDP-43, but not mouse TDP-43, in hTDP-43 mice. We also assessed the splicing patterns of the *Sort1*, *Dnajc5*, *Ppp3ca*, and *Kcnip2* gene transcripts, which are reported to be targets of TDP-43.^38^ Semi-quantitative RT-PCR analysis demonstrated that hTDP-43 mice that were injected with ASO **C1** and **15** show no differences in the splicing patterns of all tested genes at 2 weeks after injection, which were almost similar to those in WT mice (Figure S3). These results indicated that the ASO-mediated reduction in the level of human TDP-43 expression has no substantial effects on alternative splicing of the genes downstream of TDP-43 in hTDP-43 mice.

### Gapmer ASO **15** suppresses the behavioral abnormalities of hTDP-43 mice

We then analyzed whether gapmer ASO-mediated reduction of TDP-43 leads to amelioration of the abnormal phenotypes of hTDP-43 mice. For this purpose, we injected either gapmer ASO **15** or control ASO **C2** at a dose of 200 μg into the cerebral ventricles of hTDP-43 mice at 6 weeks of age, which is before the age of disease onset and the mislocalization/aggregation of TDP-43,^37^ and analyzed the therapeutic effects on these mice. To analyze the *in vivo* efficacy of ASO **15** more accurately, we newly designed the nontargeting control ASO **C2** with a scrambled sequence of ASO **15**, in which the number of each base of DNA and ENA is identical to that in ASO **15** but the sequence is scrambled (Table 2). ASO **C2** was shown to have no intrinsic target by both a human and mouse database search, and we confirmed that i.c.v. administration of ASO **C2** at a dose of 200 μg into WT mice at 6 weeks of age is well tolerated and does not cause body weight loss or detrimental effects on rotarod motor performance until 7 months of age, compared with noninjected WT mice (Figure S4). We also confirmed that the administration of ASO **C2** or ASO **15** at a dose of 200 μg into WT mice at 6 weeks of age causes minimal or no neuroinflammatory responses, as suggested by immunohistochemical analysis of microglial activation in brain slices of ASO-injected mice (Figure S5).

**Figure 3:**
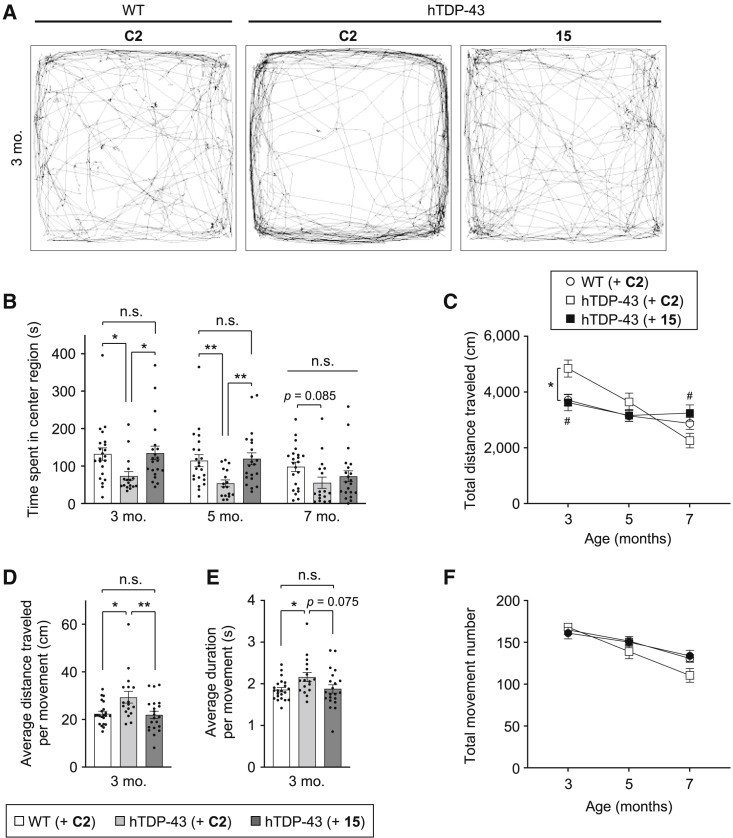
Gapmer ASO **15** improves anxiety-like behavior and abnormal locomotor functions of hTDP-43 mice in the open-field test (A–F) WT and hTDP-43 mice were injected with either control ASO **C2** or ASO **15** (200 μg) into the cerebral ventricles at 6 weeks of age, and analyzed using the open-field test at 3, 5, and 7 months of age. Tracking data of the movement of representative mice at 3 months of age are shown in (A). Time spent in the center region (B), total travel distance (C), and total movement number (F) were measured, and average travel distance (D) and duration (E) per movement were calculated and shown as bar graphs. Bar graph data are presented as the mean ± SEM of 17–22 animals in each group. Dots represent values from individual mice. Statistical analyses were performed to assess differences among groups at each time point by two-way repeated-measures ANOVA followed by the Tukey multiple comparisons test (B, C, and F) and one-way ANOVA followed by the Tukey multiple comparisons test (D and E) (∗p < 0.05, ∗∗p < 0.01, ∗∗∗p < 0.001; n.s., not significant). In (C), ∗p < 0.05: ASO **C2**-injected hTDP-43 mice versus ASO **C2**-injected WT mice; ^#^p < 0.05: ASO **15**-injected hTDP-43 mice versus ASO **C2**-injected hTDP-43 mice.

To analyze the therapeutic effects of ASO **15** on the disease phenotypes of hTDP-43 mice, including anxiety-like behavior and abnormal locomotor function, we performed the open-field test. Whereas WT mice explored the entire field of the test arena, hTDP-43 mice walked around mostly in the peripheral area of the field and rarely moved across the center region at 3 months of age (Figure 3A). In general, mice show exploratory behavior when they enter novel environments, but at the same time they tend to stay close to the periphery in unfamiliar surroundings because of their innate aversion to open spaces. This aversiveness, which can be interpreted as anxiety-like behavior, is apparently increased in hTDP-43 mice. Indeed, hTDP-43 mice injected with ASO **C2** at 6 weeks of age spent a shorter time in the center region of the field than ASO **C2**-injected WT mice at 3 months (ASO **C2**/hTDP-43 73.6 ± 11.7 s versus ASO **C2**/WT 132.3 ± 16.6 s, p < 0.05) and at 5 months (ASO **C2**/hTDP-43 54.1 ± 9.1 s versus ASO **C2**/WT 114.7 ± 16.0 s, p < 0.01) (Figure 3B), indicating increased anxiety in hTDP-43 mice. The administration of ASO **15**, however, led to substantial improvement in the anxiety-like behavior of hTDP-43 mice, as ASO **15**-injected hTDP-43 mice explored the entire field, similarly to ASO **C2**-injected WT mice (Figure 3A), and spent a longer time in the center region than ASO **C2**-injected hTDP-43 mice, both at 3 months (ASO **15**/hTDP-43 135.0 ± 18.2 s versus ASO **C2**/hTDP-43 73.6 ± 11.7 s, p < 0.05) and 5 months of age (ASO **15**/hTDP-43 120.0 ± 15.7 s versus ASO **C2**/hTDP-43 54.1 ± 9.1 s, p < 0.01) (Figure 3F). These results indicate that ASO **15** suppresses the anxiety-like behavior of hTDP-43 mice.

We also analyzed the abnormalities in locomotor function of hTDP-43 mice using the open-field test. We found that the locomotor activity of hTDP-43 mice significantly increased at an early age compared with WT mice, but subsequently decreased with age (Figure 3C). Indeed, hTDP-43 mice injected with ASO **C2** at 6 weeks of age traveled a longer distance than ASO **C2**-injected WT mice at 3 months of age (ASO **C2**/hTDP-43 4,845 ± 307 cm versus ASO **C2**/WT 3,712 ± 197 cm, p < 0.05) (Figure 3C), implying the increased locomotor activity of hTDP-43 mice. The calculated values of the average distance traveled and the average duration per movement at 3 months of age were also increased in hTDP-43 mice compared with WT mice (Figures 3D and 3E), whereas the total movement number was constant among the tested groups (Figure 3F). These data suggest that this mouse model of ALS/FTD expressing TDP-43 with an A315T mutation demonstrates hyperactivity in locomotor function at 3 months of age. This observation is in good agreement with a previous report on a TDP-43 transgenic mouse carrying the A315T mutation.^11^ However, it is noted that the hyperactive tendency of the hTDP-43 mice was not evident at later time points; ASO **C2**-injected hTDP-43 mice showed a rapid decrease in both total travel distance and a tendency of decrease in total movement number after 3 months of age, indicating a progressive decline with age in locomotor function of hTDP-43 mice (Figures 3C and 3F).

Administration of gapmer ASO **15** improved the abnormal locomotor function of hTDP-43 mice. The increased total travel distance of hTDP-43 mice due to hyperactive behavior at 3 months of age was effectively attenuated by the injection of ASO **15** at 6 weeks of age (ASO **15**/hTDP-43 3,627 ± 298 cm versus ASO **C2**/hTDP-43 4,845 ± 307 cm, p < 0.05), to a level comparable with that of ASO **C2**-injected WT mice (Figure 3C). Average scores of both the travel distance and duration per movement of hTDP-43 mice were also normalized to levels similar to those of WT mice by the injection of ASO **15** (Figures 3D and 3E). The administration of ASO **15** improved the progressive decline in spontaneous locomotor function at 7 months of age, as ASO **15**-injected hTDP-43 mice showed a significant increase in total travel distance (ASO **15**/hTDP-43 3,244 ± 291 cm versus ASO **C2**/hTDP-43 2,260 ± 263 cm, p < 0.05), which reached a level similar to that of WT mice (Figure 3C). ASO **15** treatment resulted in a tendency of improvement in the decrease in the total movement numbers of hTDP-43 mice at 5 months and 7 months of age (Figure 3F). These results indicate that ASO **15** not only effectively suppresses the hyperactivity of hTDP-43 mice observed at an early stage of disease but also improves the progressive decline in spontaneous locomotor function observed at the late stage of disease.

**Figure 4:**
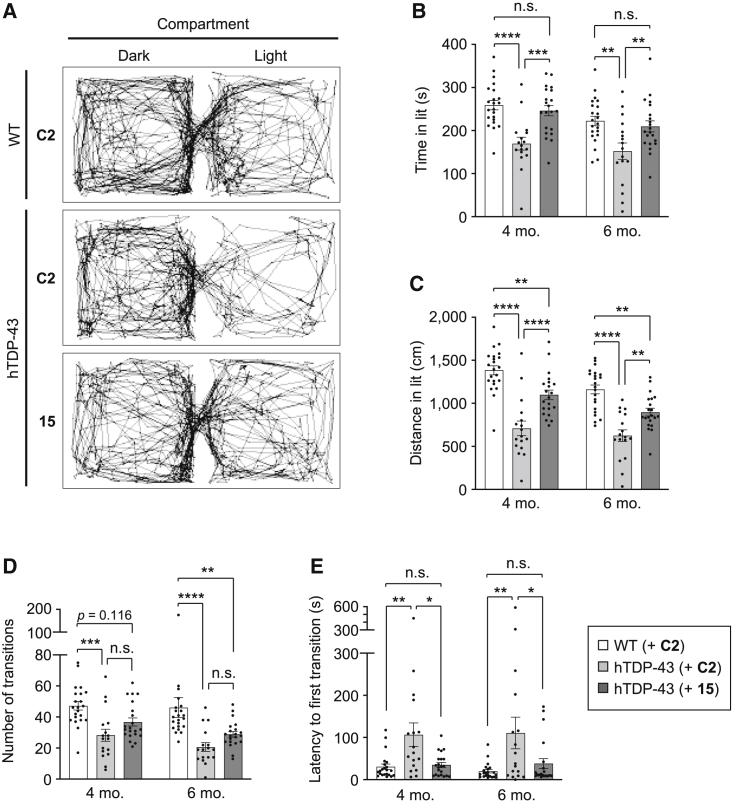
Gapmer ASO **15** improves anxiety-like behavior of hTDP-43 mice in the light and dark transition test (A–E) WT and hTDP-43 mice were injected with either control ASO **C2** or ASO **15** (200 μg) into the cerebral ventricles at 6 weeks of age, and analyzed using the light and dark transition test at 4 and 6 months of age. The representative tracking data of mouse movement at 4 months of age are shown in (A). Time spent in the light compartment (time in lit) (B), distance traveled in the light compartment (distance in lit) (C), number of transitions (D), and latency to the first transition to the light compartment (E) were measured and are shown as bar graphs. Data are presented as the mean ± SEM of 17–22 animals in each group. Statistical analyses were performed to assess differences among groups at each time point by two-way repeated-measures ANOVA followed by the Tukey multiple comparisons test (B–E). ∗p < 0.05, ∗∗p < 0.01, ∗∗∗p < 0.001, ∗∗∗∗p < 0.0001; n.s., not significant.

To further analyze the therapeutic effect of gapmer ASO **15** on the anxiety-like behavior of hTDP-43 mice, we performed the light and dark transition test. When placed in novel environments of dark and light compartments, mice preferentially explore the dark compartment because of their tendency to avoid light environments. We found that hTDP-43 mice demonstrate significantly decreased exploratory activity in the light compartment (Figure 4A), as hTDP-43 mice injected with ASO **C2** stayed for a shorter time and traveled a shorter distance in the light compartment than ASO **C2**-injected WT mice at 4 months of age (time spent in light compartment [time in lit]: ASO **C2**/hTDP-43 169.7 ± 14.9 s versus ASO **C2**/WT 258.9 ± 10.8 s, p < 0.0001; distance traveled in the light compartment [distance in lit]: ASO **C2**/hTDP-43 709 ± 85 cm versus ASO **C2**/WT 1,384 ± 53 cm, p < 0.0001) and at 6 months (time in lit: ASO **C2**/hTDP-43 152.3 ± 19.0 s versus ASO **C2**/WT 222.5 ± 11.1 s, p < 0.01; distance in lit: ASO **C2**/hTDP-43 625 ± 68 cm versus ASO **C2**/WT 1,161 ± 52 cm, p < 0.0001) (Figures 4B and 4C). Furthermore, although the total number of transitions between the two compartments was reduced, the latency to the first transition into the light compartment was substantially increased in hTDP-43 mice at 4 months (number of transitions: ASO **C2**/hTDP-43 28.2 ± 3.9 versus ASO **C2**/WT 47.2 ± 2.8, p < 0.001; latency to first transition: ASO **C2**/hTDP-43 106.8 ± 27.7 s versus ASO **C2**/WT 30.9 ± 6.4 s, p < 0.01) and 6 months of age (number of transitions: ASO **C2**/hTDP-43 20.6 ± 2.8 versus ASO **C2**/WT 46.1 ± 6.4, p < 0.0001; latency to first transition: ASO **C2**/hTDP-43 110.6 ± 37.6 s versus ASO **C2**/WT 20.0 ± 4.0 s, p < 0.01) (Figures 4D and 4E). These observations suggest that hTDP-43 mice demonstrate a strong tendency to avoid light environments compared with WT mice, indicating the increased anxiety-like behavior in hTDP-43 mice as observed in the open-field test.

We further found that the increased anxiety-like behavior of hTDP-43 mice in the light and dark transition test can be attenuated by the administration of ASO **15** (Figure 4A), as hTDP-43 mice injected with ASO **15** spent a longer time and moved a greater distance in the light compartment than control ASO **C2**-injected hTDP-43 mice, both at 4 months (time in lit: ASO **15**/hTDP-43 246.3 ± 11.9 s versus ASO **C2**/hTDP-43 169.7 ± 14.9 s, p < 0.001; distance in lit: ASO **15**/hTDP-43 1,099 ± 55 cm versus ASO **C2**/hTDP-43 709 ± 85 cm, p < 0.0001) and 6 months of age (time in lit: ASO **15**/hTDP-43 209.7 ± 12.8 s versus ASO **C2**/hTDP-43 152.3 ± 19.0 s, p < 0.01; distance in lit: ASO **15**/hTDP-43 898 ± 45 cm versus ASO **C2**/hTDP-43 625 ± 68 cm, p < 0.01), showing values similar to those of WT mice (Figures 4B and 4C). In addition, ASO **15-**administered hTDP-43 mice showed an increased tendency in the number of transitions compared with **C2**-administered mice (Figure 4D), and also demonstrated a marked reduction in the latency to the first transition to the light compartment, both at 4 months (ASO **15**/hTDP-43 35.0 ± 5.8 s versus ASO **C2**/hTDP-43 106.8 ± 27.7 s, p < 0.05) and 6 months of age (ASO **15**/hTDP-43 38.4 ± 11.8 s versus ASO **C2**/hTDP-43 110.6 ± 37.6 s, p < 0.05) (Figure 4E), demonstrating a significant increase in exploratory activity in the light environment. Taken together, these results demonstrate that the reduction in TDP-43 levels by the injection of gapmer ASO **15** leads to sustained improvement of the abnormal phenotypes of hTDP-43 mice that are caused by the expression of human TDP-43 with an A315T mutation, including anxiety-like behavior and the progressive decline in locomotor function.

### Gapmer ASO **15** does not improve the grip strength but normalizes the body weight increase of hTDP-43 mice

**Figure 5:**
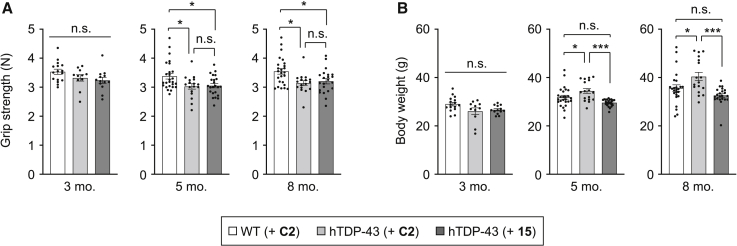
Gapmer ASO **15** normalizes the increase in body weight but not the decrease in grip strength of hTDP-43 mice WT and hTDP-43 mice were injected with either control ASO **C2** or ASO **15** (200 μg) into the cerebral ventricles at 6 weeks of age, and grip strength (A) and body weight (B) were measured at 3, 5, and 8 months of age. Data are presented as the mean ± SEM of 11–26 animals in each group. Statistical analyses were performed to assess differences among groups using one-way ANOVA followed by the Tukey multiple comparisons test. ∗p < 0.05, ∗∗∗p < 0.001; n.s., not significant.

We also analyzed the effects of ASO administration on the grip strength abnormality of hTDP-43 mice. The reduced grip strength of hTDP-43 mice compared with WT mice becomes evident by 5 months of age, and the administration of ASO **15** into hTDP-43 mice at 6 weeks of age appeared to improve this weakness at 5 months and 8 months of age, when the grip strength scores were normalized by body weight (Figure S6). However, we found that the original scores of grip strength without body weight normalization were unchanged between ASO **C2**-injected and ASO **15**-injected hTDP-43 mice (Figure 5A), implying that ASO **15** may simply be improving the body weight abnormality rather than the grip strength of hTDP-43 mice. Indeed, hTDP-43 mice that were injected with control ASO **C2** had slightly higher body weights than ASO **C2**-injected WT mice at 5 months (ASO **C2**/hTDP-43 34.5 ± 4.2 g versus ASO **C2**/WT 31.9 ± 4.1 g, p < 0.05) and 8 months of age (ASO **C2**/hTDP-43 40.4 ± 7.2 g versus ASO **C2**/WT 35.7 ± 6.8 g, p < 0.05), but not at 3 months of age (Figure 5B). The administration of gapmer ASO **15** into hTDP-43 mice significantly attenuated this body weight increase both at 5 months (ASO **15**/hTDP-43 29.6 ± 1.4 g versus ASO **C2**/hTDP-43 34.5 ± 4.2 g, p < 0.001) and 8 months of age (ASO **15**/hTDP-43 32.6 ± 3.5 g versus ASO **C2**/hTDP-43 40.4 ± 7.2 g, p < 0.001), resulting in the normalization of the body weights of hTDP-43 mice to levels similar to those of ASO **C2**-injected WT mice, with no statistically significant difference between the two groups (Figure 5B). These results demonstrate that gapmer ASO **15** effectively ameliorates the abnormal increase in body weight of hTDP-43 mice but not the reduced grip strength under the tested condition.

### Gapmer ASO **15** suppresses the mislocalization and cytoplasmic aggregation of TDP-43 in hTDP-43 mice

**Figure 6:**
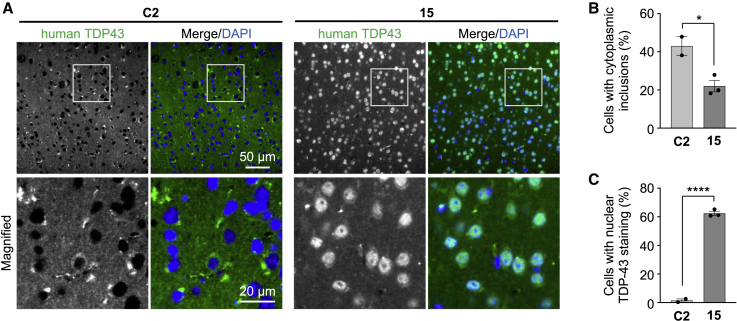
Gapmer ASO **15** suppresses the formation of cytoplasmic aggregates and mislocalization of TDP-43 in hTDP-43 mice (A) hTDP-43 mice were injected with either control ASO **C2** or ASO **15** (200 μg) into the cerebral ventricles at 6 weeks of age and analyzed by immunohistochemistry using an antibody against human TDP-43 at 18 months of age. Representative images are shown, and magnified images of the boxed region are shown in the bottom panels. DAPI was used for nuclear staining. Scale bars, 50 μm (top panels) and 20 μm (bottom panels). (B and C) Bar graphs showing the ratios of cells with cytoplasmic inclusions (B) and the nuclear localization of TDP-43 (C), calculated from the microscopic data in (A). Data in (B) and (C) are presented as the mean ± SEM of 2–3 animals in each group. Statistical analyses were performed to assess differences from the control group by Student’s t test. ∗p < 0.05, ∗∗∗∗p < 0.0001.

We also investigated whether the ASO-mediated reduction in TDP-43 level affects the TDP-43 pathology of hTDP-43 mice. A previous report demonstrated that hTDP-43 mice with an A315T mutation demonstrates mislocalization and cytoplasmic aggregation of TDP-43 in the spinal cord at 10 months of age, which can be specifically detected by a human-specific monoclonal antibody against TDP-43.^37^ Although we confirmed the presence of TDP-43 pathology in the spinal cord as well as in the cortex and hippocampus of hTDP43 mice at 10 months of age, the rate of mislocalization and the formation of cytoplasmic inclusions of TDP-43 were found to be relatively low under our experimental conditions (<5% of cells). However, we found that TDP-43 pathology becomes prominent with further aging, as TDP-43 was almost absent in the nucleus but was aggregated as inclusions in the cytoplasm in cerebral cortical neurons of hTDP-43 mice at 18 months of age (Figure 6A). Surprisingly, the administration of ASO **15** substantially changed the cellular distribution of TDP-43, as hTDP-43 mice injected with ASO **15** at 6 weeks of age showed a dominantly nuclear localization of TDP-43 with reduced cytoplasmic aggregates at 18 months of age, compared with ASO **C2**-injected hTDP-43 mice, which showed a large amount of cytoplasmic aggregates (Figures 6B and 6C). These results demonstrate that lowering the level of TDP-43 by ASO administration leads to sustained therapeutic effects that prevent the mislocalization and cytoplasmic aggregation of TDP-43 in hTDP-43 mice.

## Discussion

Although TDP-43 plays central roles in the pathogenesis of ALS/FTD, targeting TDP-43 itself has been considered to be inappropriate because TDP-43 is crucial for various cellular functions.^15^ Alternatively, efforts have been focused on identifying other strategies that lower TDP-43 toxicity indirectly while maintaining its cellular functions.^18^^,^^39^ This is one of the reasons why therapeutic approaches directly targeting TDP-43 using ASOs have not been investigated to date and, therefore, it was unclear whether TDP-43 is an effective or appropriate target for ASO-based therapies against ALS/FTD as well as other TDP-43 proteinopathies. The present study addressed this issue by developing novel ENA-modified gapmer-type ASOs that target human TDP-43 and testing their therapeutic potential using a mouse model of ALS/FTD that develops TDP-43 pathology. We demonstrated that i.c.v. administration of a TDP-43-targeting ASO into hTDP-43-expressing mice efficiently lowers TDP-43 levels in the brain and spinal cord, leading to the suppression of both disease phenotypes and TDP-43 pathology. These results demonstrate that TDP-43 is an effective therapeutic target for ALS/FTD, indicating the possibility of the establishment of disease-modifying therapies using TDP-43-targeting ASOs that would suppress the onset or delay the progression of TDP-43 proteinopathies, including ALS/FTD.

We surprisingly found that a single administration of TDP-43-targeting ASOs through the cerebral ventricles provided unexpected long-lasting therapeutic effects in hTDP-43 mice. These therapeutic effects of the TDP-43-targeting ASOs were observed even 4–6 months post injection on various disease phenotypes, including anxiety-like behavior and abnormal locomotor functions (Figure 3, Figure 4 and 4). Most importantly, hTDP-43 mice that were injected with ASOs at 6 weeks of age demonstrated preferential nuclear localization and reduced cytoplasmic aggregates of TDP-43 in cortical neurons at 18 months of age (Figure 6), indicating the long-term therapeutic effects of TDP-43-targeting ASOs on brain pathology. One possible reason for these sustained effects of ASOs may be attributed to the use of ENA, which is a modified nucleic acid with an ethylene bridge.^25^ Because ENA has higher nuclease resistance than other modified nucleic acids, such as BNA/LNA, ENA-modified ASOs are expected to have higher stability against degradation, enabling prolonged suppression of TDP-43 expression. However, our data on the validation of TDP-43 knockdown in ASO-injected hTDP-43 mice showed that the suppression of TDP-43 expression in the brain was sustained for at least 12 weeks after injection (Figure 2) but was no longer evident 24 weeks after injection (Figure S1). In general, ASOs are not expected to be active for more than half a year after injection; hence, repeated administration is widely applied for long-term therapeutic approaches using ASOs. Therefore, our data showed an apparent discrepancy between the duration of TDP-43 knockdown and the duration of the therapeutic improvement of disease phenotypes and brain pathology, which cannot be fully explained by the higher stability of ENA. Interestingly, similar sustained therapeutic effects of ASOs have been demonstrated in a previous report in which the infusion of huntingtin-targeting ASOs into the lateral ventricle of HD mice for 2 weeks resulted in sustained phenotypic reversal for at least 4 months, even after huntingtin protein levels had returned to the initial levels.^40^ Neurodegenerative diseases, such as ALS/FTD and HD, are mostly late-onset diseases showing progressive symptoms, whereas the disease-causative proteins, such as TDP-43 and mutant huntingtin, are constitutively expressed from birth. This implies that not the expression of disease proteins at a certain point in time but the long-term accumulation of abnormal species of disease-causative proteins that are formed over a long period of time, including misfolded monomers, oligomers, and aggregates, may be more closely associated with neuronal dysfunction and disease phenotypes in patients. We hence speculate that the reduction in the expression level of TDP-43 by TDP-43-targeting ASOs effectively delays or prevents the formation of abnormal aggregates of TDP-43, leading to long-lasting therapeutic suppression of behavioral deficits and brain pathology in hTDP-43 mice, even after ASO-mediated TDP-43 knockdown has ended. It remains to be clarified as to how the sustained effects of ASOs are actually achieved and how long this effect can be sustained after ASO injection. Nevertheless, our findings suggest the possibility that a transient reduction in TDP-43 level by the ASO-mediated approach can lead to long-lasting therapeutic effects in patients with ALS/FTD.

Another important finding of this study is that the transient reduction of TDP-43 prevented TDP-43 pathology, including the mislocalization and cytoplasmic aggregation of TDP-43 (Figure 6). Although further verification using biochemical or other experimental methods are needed, this finding suggests that the cellular level of TDP-43 is likely to be a factor that regulates or affects the intracellular behavior of TDP-43 *in vivo*, i.e., the cellular localization and the aggregation propensity of TDP-43. The cellular level of TDP-43 is tightly regulated by a negative feedback mechanism to maintain a constant expression level.^28^ However, this tight regulation might be impaired in the disease state,^30^ as patients with sporadic ALS have been demonstrated to have increased TDP-43 expression in the spinal cord at both the mRNA and protein levels by 2.5-fold and 1.8-fold, respectively, compared with controls.^34^ Transgenic animals overexpressing WT TDP-43 demonstrated dose-dependent cytoplasmic accumulation of TDP-43 aggregates with pathological modifications of phosphorylation and ubiquitination.^2^^,^^3^ Thus, the TDP-43-targeting approach is expected to normalize the abnormal intracellular behavior of TDP-43 by lowering its expression level, which might be abnormally regulated in the disease state, although other factors, such as missense mutations in TDP-43 and dysfunction in intracellular and nucleocytoplasmic transport,^41^^,^^42^ also play an important role in abnormal TDP-43 behavior and disease pathogenesis.

Our study suggests that lowering the level of disease-causative human TDP-43 suppresses behavioral abnormalities and TDP-43 pathology, leading to therapeutic benefits in hTDP-43 mice. However, we cannot exclude the possibility that the potential changes in the level of endogenous mouse TDP-43 might be involved in the therapeutic benefits observed in hTDP-43 mice injected with gapmer ASOs. Because TDP-43 expression is tightly regulated by an autoregulatory mechanism, the ASO-mediated suppression of human TDP-43 expression might affect mouse TDP-43 expression, which may indirectly contribute to the beneficial outcomes in hTDP-43 mice. Nevertheless, experimental evidence that neither the expression of mouse TDP-43 (Figure S2) nor the cryptic splicing of downstream genes that are regulated by TDP-43 (Figure S3) are affected by the administration of ASO **15** suggests that endogenous mouse TDP-43 is not involved in the therapeutic effects observed in the present study, which strongly supports our conclusion.

ASOs targeting disease-causative genes associated with ALS/FTD, including *SOD1*, *C9orf72*, *ataxin-2*, and *FUS*, have already been demonstrated to show therapeutic effects in preclinical evaluations and are now being clinically tested in patients, holding great promise for the development of ASO-based therapies for ALS/FTD in the near future. We further extended this by demonstrating the therapeutic effects of a TDP-43-lowering approach using an ENA-modified ASO. Because TDP-43 plays a central role in the pathogenesis of ALS/FTD, our TDP-43-targeting approach has great potential for treating most patients with ALS/FTD as well as those with other TDP-43 proteinopathies. Future studies to validate the efficacy of the TDP-43-targeting approach in other models and to investigate the pathological and phenotypic reversal of disease by injecting ASOs at later time points, particularly after disease onset, would provide a better understanding of the therapeutic potential of this approach. Furthermore, it may be worthwhile to investigate the efficacy of the TDP-43-lowering strategy using other approaches, including RNAi-mediated knockdown using siRNA or microRNA, and the inhibition of splicing and the translation of TDP-43 mRNA using blocker ASOs with modified nucleic acids or morpholino backbones.

### Limitations of the study

Although our data provide the first preclinical proof of concept for the TDP-43-targeting approach using gapmer ASOs for the treatment of ALS/FTD, a limitation of this study is the lack of data on the toxic side effects that might result from the reduction of TDP-43. hTDP-43 mice express not only human TDP-43 but also endogenous mouse TDP-43, which is not targeted by gapmer ASOs. Therefore, our experimental design mainly focuses on the efficacy of the TDP-43-lowering approach using ASOs. Because TDP-43 is important for cellular activities associated with RNA transport and processing as well as RNA granule formation, and the deletion of TDP-43 causes embryonic lethality in mice,^14^^,^^15^^,^^27^^,^^43^ the complete inhibition of TDP-43 may result in the loss of TDP-43-associated cellular functions, leading to deleterious consequences for cell survival. Therefore, further studies of the safety issues associated with TDP-43 reduction, together with efforts to optimize the design of ASOs for minimizing their potential toxicity *in vivo*, are needed to promote the therapeutic development for clinical use.

## Materials and methods

### ASO synthesis

Modified oligonucleotides containing 2′-*O*,4′-*C*-ethylene nucleosides were prepared by solid-phase phosphoramidite chemistry using DNA/RNA synthesizer NTS H-6 (Nihon Techno Service). Reagents for ASO synthesis were purchased from Glen Research. The coupling of 2′-*O*,4′-*C*-ethylene nucleoside-3′-phosphoramidite was performed according to standard synthesis cycles, except for an elongation of the coupling time (15 min). After synthesis, the resins were treated with concentrated aqueous ammonia at 55°C for 8 h. The crude products were purified by C18 silica gel column chromatography with a gradient of CH_3_CN (YMC-Triart C18 [YMC, Japan], 4.6 × 150 mm, 100 mM triethylammonium acetate [pH 7.0]), treated with 10% acetic acid in H_2_O for 8 h, and then purified by reverse-phase high-performance liquid chromatography with a gradient of CH_3_CN (YMC-Triart C18, 0.1 M triethylammonium acetate [pH 7.0]).

### Cell-culture experiments

HEK293 cells were grown and maintained in DMEM supplemented with 10% (v/v) fetal bovine serum. For western blotting analysis, HEK293 cells were transfected with gapmer ASOs using Lipofectamine RNAiMAX reagent (Thermo Fisher Scientific), incubated for 48 h, and lysed with RIPA buffer (Nacalai Tesque). Lysed solutions were collected and centrifuged to remove cell debris, resulting in clear cell lysate solutions. The protein content of the cell lysates was estimated using a DC Protein Assay kit (Bio-Rad). Proteins in cell lysates were separated using 5%–20% gradient SDS-PAGE gels (ATTO) and transferred onto polyvinylidene fluoride (PVDF) membranes (Bio-Rad). The membranes were incubated overnight with the following antibodies at 4°C: anti-TDP-43 (10782-2-AP rabbit polyclonal; Proteintech), and anti-β-actin (AC-40 mouse monoclonal; Sigma). As secondary antibodies, horseradish peroxidase (HRP)-conjugated immunoglobulins (IgGs) were used. The HRP signal was visualized with ImmunoStar Zeta chemiluminescence solution (Fujifilm Wako) and captured with an Amersham Imager 600 CCD imaging system (Cytiva). Acquired images were analyzed using ImageJ software. For qRT-PCR analysis, HEK293 cells were transfected with ASOs and incubated for 48 h as above, and total RNA was extracted using TRIzol reagent (Thermo Fisher Scientific). cDNA was synthesized from total RNA using a QuantiTect Reverse Transcription Kit (QIAGEN), and real-time PCR was performed using the CFX96 Touch Real-Time PCR System (Bio-Rad) and TB Green Premix Ex Taq II (Takara). The primer sequences are as follows: *TDP-43* forward, 5′-GGG AAA TCT GGT GTA TGT TGT C-3′; *TDP-43* reverse, 5′-TTT CAG GTC CTG TTC GGT TG-3′; *GAPDH* forward, 5′-AAG GTG AAG GTC GGA GTC AAC-3′; *GAPDH* reverse, 5′-GGG GTC ATT GAT GGC AAC AAT A-3′. For the cell viability assay, HEK293 cells were transfected with ASOs and incubated for 48 h as above. After incubation, the cells were treated with WST-1 reagent (Takara) for 2 h, and cell viability was analyzed according to the manufacturer’s instructions. All experiments were performed independently at least three times.

### Mice and ASO injections

All mouse experiments were approved by the Animal Experiment Committee of Osaka University and Nagoya University, and were performed in accordance with their guidelines on animal experiments. hTDP-43 mice were generated by the Julien laboratory and have been described previously.^37^ Mice were housed on a 12:12-h light/dark cycle, with food and water provided *ad libitum*. For ASO administration, mice at 6 weeks of age were stereotactically injected with 5 μL of ASO in saline (a total of 200 μg) into the right ventricle using a 5-μL syringe (Hamilton). The coordinates for injection were 2 mm anterior to the lambdoid suture, 1 mm lateral from the sagittal suture, and a depth of 2–3 mm.

### Mouse phenotype analyses

Body weight, grip strength, and rotarod performance were assessed as described previously.^44^ The open-field test was performed at 3, 5, and 7 months of age using an open-field arena (60 × 60 × 30 cm). Mice were first placed in the center area of the arena, and their spontaneous locomotor movements were tracked and recorded for a total of 10 min. The total distance that the mice traveled and the time they spent in the center region of the arena were analyzed. The center region was defined as nine sections (3 × 3) at the center of the arena when the arena is divided into 25 equal square sections (5 × 5). The light and dark transition test was performed at 4 and 6 months of age using a box (42 × 20 × 25 cm) that was divided into two compartments of equal size. One compartment was brightly illuminated whereas the other one was dark. The apparatus had an opening (5 × 3 cm) with a sliding door in the middle of the wall joining the two compartments. The sliding door was opened 3 s after the mice were placed into the dark compartment. Mice were allowed to move freely between the two compartments for 10 min, and their behavior was recorded by a camera installed on the ceiling of each compartment. The distance traveled in each compartment, the total number of light-dark transitions, the time spent in each compartment, and the latency to enter the light compartment were analyzed. Although rotarod motor performance has been reported to be reduced in hTDP-43 mice at 8 months of age,^37^ this was not confirmed under our experimental conditions (Figure S7), possibly due to a reduction in the expression levels of the TDP-43 transgene in this mouse line, as suggested by the report from Julien’s group.^45^

### Immunohistochemical analyses

Anesthetized mice were perfused with PBS followed by 4% paraformaldehyde fixative in phosphate buffer through the left cardiac ventricle. Brains were removed after perfusion, post-fixed in 10% phosphate-buffered formalin, and processed for paraffin embedding. Brain sections of 3 μm thickness were deparaffinized and blocked in PBS containing 2% BSA and 0.5% Triton X-100 for 1 h at room temperature. The sections were then incubated with an anti-TDP-43 antibody (60019-2-Ig mouse monoclonal, Proteintech, 1:500), or an anti-Iba-1 antibody (019-19741 rabbit polyclonal, Wako, 1:1,000) at 4°C overnight, followed by an Alexa Fluor 488-conjugated goat IgG (Invitrogen, 1:1,000) for 1 h at room temperature. The sections were mounted with VECTASHIELD HardSet antifade mounting medium (Vector Laboratories) and analyzed using a fluorescence microscope (BZ-X800; Keyence). The formation of cytoplasmic inclusions and the nuclear localization of hTDP-43 in the cerebral cortex were quantitatively analyzed (∼300 neuronal cells per image, a total of ∼900 cells per mouse).

### Biochemical analyses of mouse tissues

For immunoblotting of hTDP-43 in hTDP-43 mouse tissues, the brain cortex, hippocampus, and spinal cord were homogenized in RIPA buffer containing protease inhibitor cocktail (Nacalai Tesque) and centrifuged at 15,000 × *g* for 10 min at 4°C, after which the supernatants were collected. Proteins were separated using 5%–20% gradient SDS-PAGE gels and transferred to PVDF membranes. The membranes were incubated overnight with the following primary antibodies at 4°C: anti-hTDP-43 (H00023435-M01 mouse monoclonal [clone 2E2-D3]; Abnova), and anti-β-actin (AC-40 mouse monoclonal; Sigma). As secondary antibodies, HRP-conjugated IgGs were used. The HRP signal was visualized by ImmunoStar Zeta chemiluminescence solution and captured using an Amersham Imager 600 CCD imaging system. Acquired images were analyzed using ImageJ software. For qRT-PCR analysis, total RNA was extracted from the brain cortex, hippocampus, and spinal cord using TRIzol reagent (Thermo Fisher Scientific). cDNA was synthesized from total RNA using a QuantiTect Reverse Transcription Kit (QIAGEN), and real-time PCR was performed using the CFX96 Touch Real-Time PCR System (Bio-Rad) and TB Green Premix Ex Taq II (Takara). For semi-quantitative RT-PCR analysis, cDNA was synthesized from total RNA as above, and PCR was performed using PCR Thermal Cycler Dice Touch (Takara). The resultant PCR fragments were analyzed by 5%–20% gradient PAGE gels (ATTO). The primer sequences used were as follows: mouse *TDP-43* forward, 5′-ATT TGA GTC TCC AGG TGG GTG TGG-3′; mouse *TDP-43* reverse, 5′-GTT TCA CTA TAC CCA GCC CAC TTT TCT TAG G-3′; *Actin* forward, 5′-CGT GCG TGA CAT CAA AGA GAA-3′; *Actin* reverse, 5′-CAA TAG TGA TGA CCT GGC CGT-3′; *Sort1* forward, 5′-ATG AAT CCC GCC AGA GAA G-3′; *Sort1* reverse, 5′-GAC AAG CAT CAG TCC CAC GA-3′; *Dnajc5* forward, 5′-ACC TGA GGG TGA GGA GAC AG-3′; *Dnajc5* reverse, 5′-GGC TGT ATG ACG ATC GGT GT-3′; *Ppp3ca* forward, 5′-CTC TCT GGC GGG AAA CAG AC-3′; *Ppp3ca* reverse, 5′-GAG GCG AGA GCC TTG TTG AT-3′; *Kcnip2* forward, 5′-AAT CCC GAG ATT TGG ACG GC-3′; *Kcnip2* reverse, 5′-TGG CAC ACC GTG GAT AGT TC-3′.

### Statistical analyses

Western blotting and cell viability data were analyzed by one-way analysis of variance (ANOVA) followed by the Dunnett’s multiple comparison test. qRT-PCR data from the cell experiments and the knockdown efficiency and immunohistochemistry data from mice were analyzed by Student’s t test. Data from body weight, grip strength, the open-field test, and the light/dark transition test of hTDP-43 mice were analyzed either by one-way ANOVA followed by the Tukey multiple comparisons test or by two-way repeated-measures ANOVA followed by the Tukey multiple comparisons test. For all analyses, GraphPad Prism (GraphPad Software) was used. A p value of less than 0.05 was considered to indicate a statistically significant difference between groups.

## Data Availability

All data related to this study are available upon request.

## Supplemental information

Document S1. Figures S1–S8

Document S2. Article plus supplemental information
