# Supplementary material for: Sustained therapeutic benefits by transient reduction of TDP-43 using ENA-modified antisense oligonucleotides in ALS/FTD mice
Source: Mol Ther Nucleic Acids. 2023 Jan 16;31:353–66. doi: 10.1016/j.omtn.2023.01.006 (PMC9925842; doi:10.1016/j.omtn.2023.01.006)
Supplement: Document S1. Figures S1–S8 [file mmc1.pdf]

OMTN, Volume *31*

Supplemental information

Sustained therapeutic benefits by transient reduction of TDP-43 using ENA-modified antisense oligonucleotides in ALS/FTD mice

Toshihide Takeuchi, Kazuhiro Maeta, Xin Ding, Yukako Oe, Akiko Takeda, Mana Inoue, Seiichi Nagano, Tsuyoshi Fujihara, Seiji Matsuda, Shinsuke Ishigaki, Kentaro Sahashi, Eiko N. Minakawa, Hideki Mochizuki, Masahiro Neya, Gen Sobue, and Yoshitaka Nagai

Figure S1

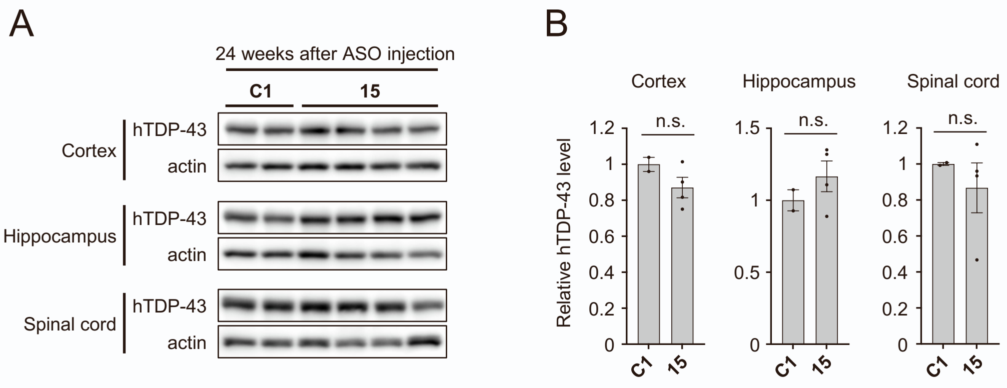

Figure S1. Gapmer ASO 15 shows no detectable reduction of TDP43 in the brain and spinal cord of hTDP-43 mice at 24 weeks after injection
Western blot images (**A**) and bar graphs (**B**) showing relative hTDP-43 levels in hTDP-43 mice that were injected with either control ASO **C1** or ASO **15** (200 µg) into the cerebral ventricles at 6 weeks of age. Protein levels of hTDP-43 in the cortex, hippocampus, and spinal cord were analyzed at 24 weeks after ASO injection. Actin was used as a loading control. Data are presented as the mean ± SEM of 2 to 4 animals. Statistical analyses were performed to assess differences from the control group by the Student *t*-test (n.s., not significant).

Figure S2

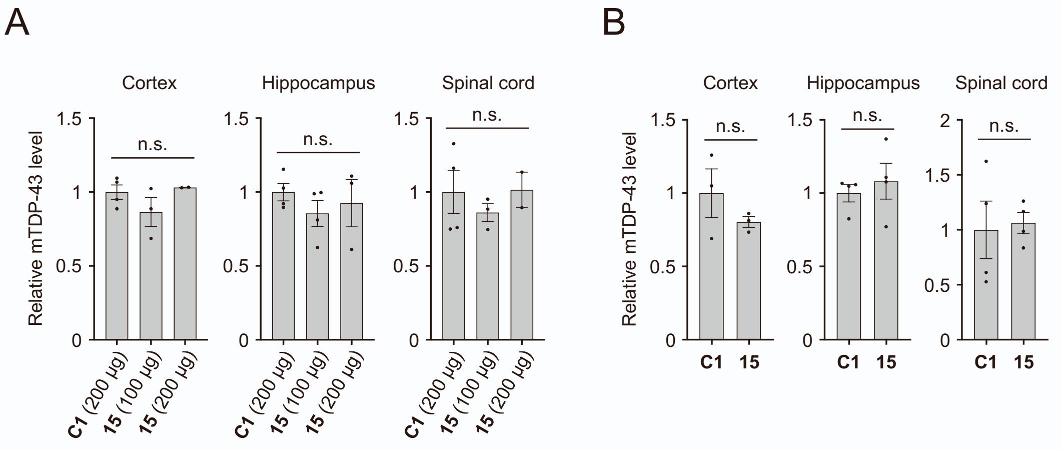

Figure S2. Administration of gapmer ASO 15 does not affect the expression of endogenous mouse TDP43 in the brain and spinal cord of hTDP-43 mice at 2 weeks and 12 weeks after injection

(**A-B**) Bar graphs showing mRNA levels of endogenous mouse TDP-43 (mTDP-43) in hTDP-43 mice that were injected with either control ASO **C1** (200 µg) or ASO **15** (**A**: 100 µg or 200 µg; **B**: 200 µg) into the cerebral ventricles at 6 weeks of age, measured by quantitative RT-PCR analysis. mRNA levels of mTDP-43 in the cortex, hippocampus, and spinal cord were analyzed at 2 weeks (**A**) and 12 weeks (**B**) after ASO injection. The mRNA levels of β-actin were also measured and used for normalization. Data are presented as the mean ± SEM of 2 to 4 animals. Statistical analyses were performed to assess differences from the control group by one-way ANOVA followed by the Dunnett multiple comparison test in **A,** and the Student *t*-test in **B** (n.s., not significant).

Figure S3

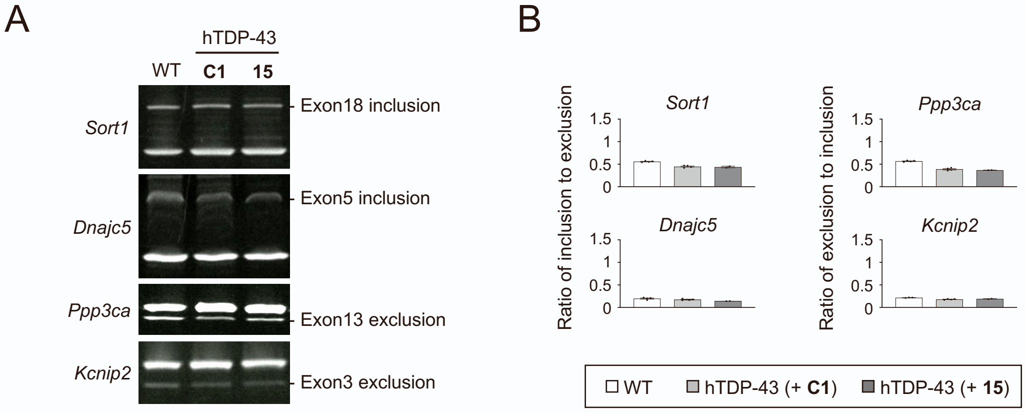

Figure S3. Administration of gapmer ASO 15 does not affect the splicing patterns of genes downstream of TDP43

(**A**) Representative PAGE images of semiquantitative RT-PCR products from brain samples of WT mice, or hTDP-43 mice that were injected with gapmer ASO **C1** or ASO **15** (200 μg) into the cerebral ventricles at 6 weeks of age. Changes in splicing patterns of selected genes that have been reported to be targets of TDP-43 (Polymenidou *et al.*, ***Nat Neurosci*** 2011) were analyzed at 2 weeks after ASO injection. Alternatively spliced RNAs including (*Sort1* and *Dnajc5*) or excluding exons (*Ppp3ca* and *Kcnip2*) have been reported to increase upon TDP-43 depletion. (**B**) Bar graphs showing the ratios of PCR products in **A**. Data are presented as the mean ± SEM of 2 to 4 mice.

Figure S4

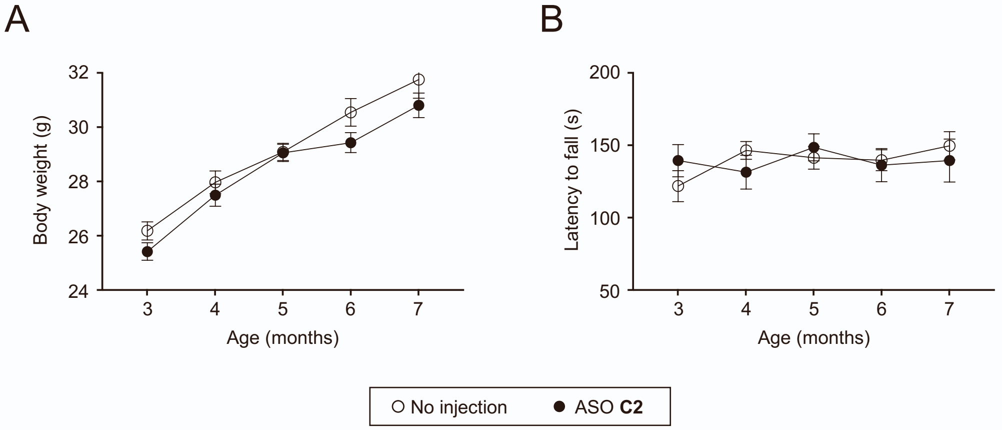

Figure S4. Gapmer ASO C2 does not cause body weight loss or abnormalities in motor performance in WT mice

WT mice were injected with either control ASO **C2** (200 μg) at 6 weeks of age, and body weight (**A**) and motor performance using the rotarod test (**B**) were analyzed from 3 to 7 months of age. WT mice without ASO injection were used as a negative control. Data are presented as the mean ± SEM of 9 to 11 mice in each group. Statistical analyses were performed to assess differences between individual groups at each time point using two-way repeated measures ANOVA followed by the Bonferroni multiple comparison test. No statistical differences were detected at any time point in body weight (**A**) and motor performance (**B**).

Figure S5

A

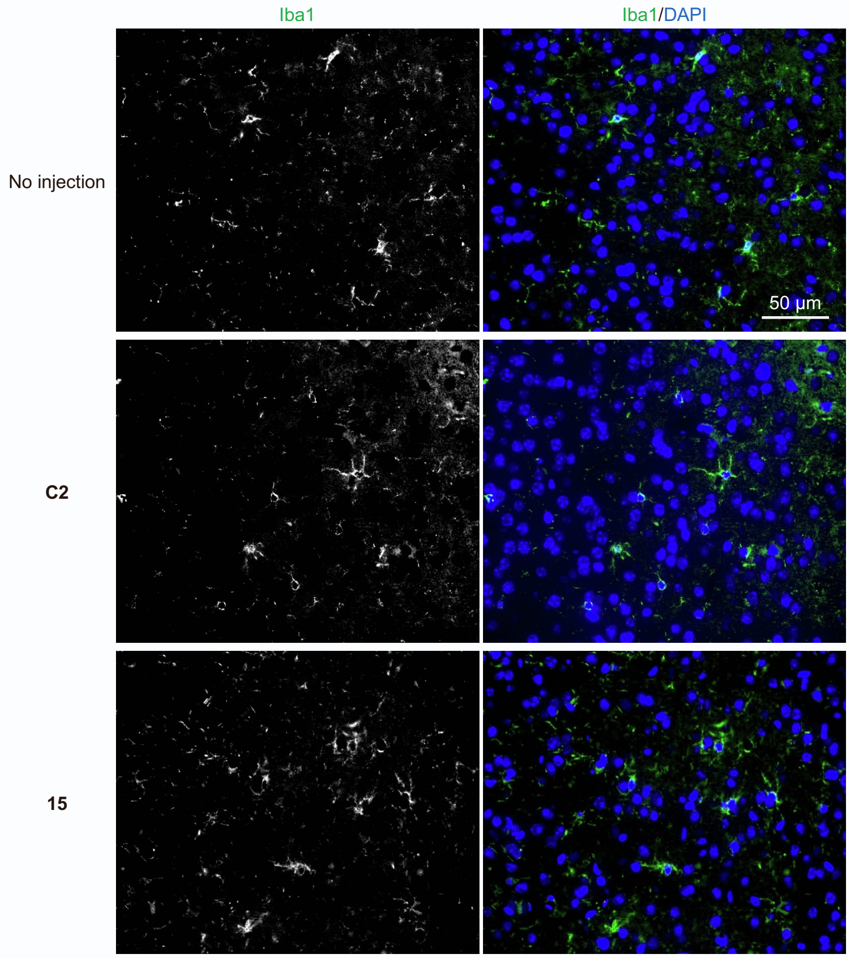

Figure S5. ASO injection does not cause microglial activation
WT mice were injected with either the control ASO **C1** or ASO **15** (200 μg) at 6 weeks of age, and analyzed by immunohistochemistry using an antibody against Iba-1 at 2 weeks after injection. Representative images are shown. DAPI was used for nuclear staining. Scale bar: 50 μm

Figure S6

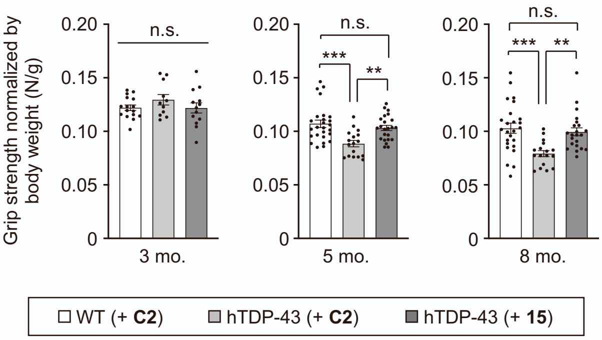

Figure S6. Gapmer ASO 15 improves grip strength normalized by body weight in hTDP-43 mice

Grip strength (**Figure 5A**) were normalized by the body weight of each mouse (**Figure 5B**), and are shown as bar graphs. Data are represented as the mean ± SEM of 11 to 26 mice in each group. Statistical analyses were performed to assess differences among the groups by one-way ANOVA followed by the Tukey multiple comparison test (***p* < 0.01, ****p* < 0.001; n.s., not significant).

Figure S7

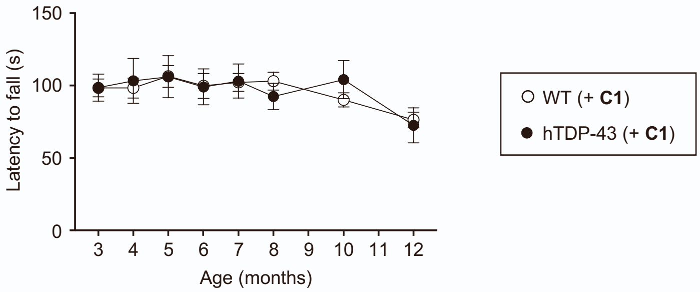

Figure S7. hTDP-43 mice do not show abnormalities in rotarod performance
WT and hTDP-43 mice were injected with the control ASO **C1** (200 µg) at 6 weeks of age, and motor performance was analyzed using the rotarod test from 3 to 12 months of age. Data are presented as the mean ± SEM of 8 to 16 mice in each group. Statistical analyses were performed to assess differences between individual groups at each time point using two-way repeated measures ANOVA followed by the Bonferroni multiple comparison test. No statistically significant differences in motor performance were detected at any time point.

Figure S8

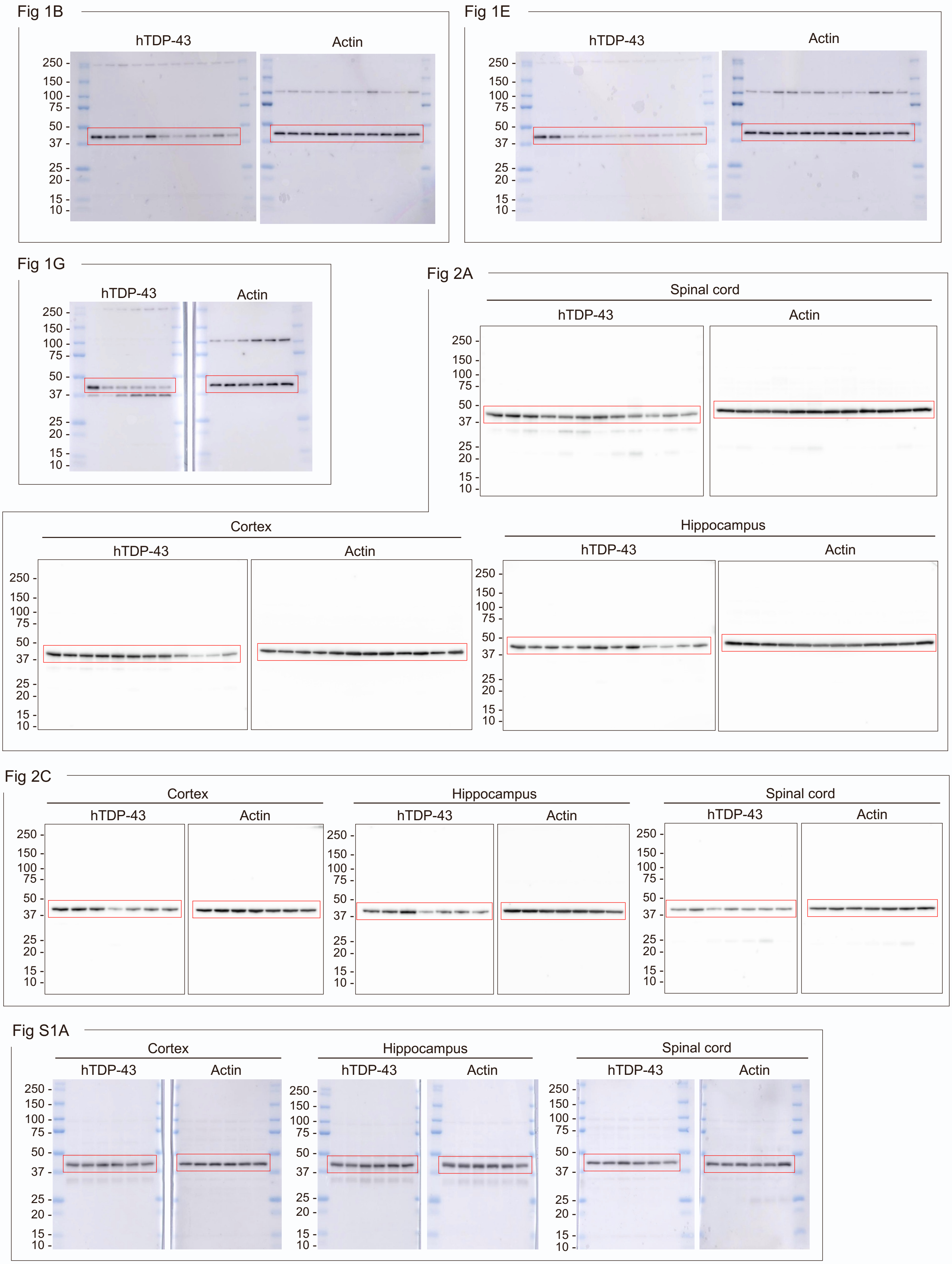

Figure S8. Uncropped images of the Western blot analyses performed in this study
